# Short-term use of CGM in youth onset type 2 diabetes is associated with behavioral modifications

**DOI:** 10.3389/fendo.2023.1182260

**Published:** 2023-05-29

**Authors:** Jacquelyn Manfredo, Tyger Lin, Radhika Gupta, Kai Abiola, Margaret West, Kelly Busin, Julia Tracey, Elizabeth A. Brown, Sheela N. Magge, Risa M. Wolf

**Affiliations:** Department of Pediatrics, Division of Endocrinology, Johns Hopkins University School of Medicine, Baltimore, MD, United States

**Keywords:** adolescents, barriers, continuous glucose monitors, glycemic control, type 2 diabetes, youth

## Abstract

**Background:**

Continuous glucose monitoring (CGM) is beneficial to glycemic control in youth with type 1 diabetes (T1D) and adults with type 2 diabetes (T2D); however, studies in youth with T2D are limited.

**Objective:**

Determine if 10-day trial CGM use in youth with T2D improves glycemic control and behavioral modifications.

**Methods:**

Youth with T2D > 3 months, on insulin, with no prior CGM use were enrolled. Staff placed CGM and provided education. Participants received 5-day and 10-day follow-up phone calls to review CGM data, behavioral modifications, and adjust insulin doses as needed. We compared 5-day to 10-day TIR, and baseline to 3-6 month HbA1c via paired t-test.

**Results:**

Participants (n=41) had median age of 16.2 y, were 61% female, 81% NH Black, median diabetes duration of 0.8 y, and baseline HbA1c of 10.3%. A majority had household income<$50,000 (81%) and parental education level of HS or less (73%). Average 5-day TIR 49% was similar to 10-day TIR 51% (p=0.62). There was no change in HbA1c after 3-6 months (10.2% v 10.3%, p=0.89). Nineteen participants completed full 10-day CGM use; of those, 84% wanted a CGM long-term. Adolescents reported behavioral changes including increased blood sugar checks, increased insulin administration and overall improved diabetes management.

**Conclusion:**

Although 10-day CGM use did not impact short-term or long-term glycemic control in youth with T2D, most participants reported behavioral changes and wanted to continue using CGM. Future studies with longer use of CGM may clarify the potential impact of CGM in youth with T2D.

## 1 Introduction

The prevalence of youth-onset type 2 diabetes (T2D) is on the rise, especially in minority and disadvantaged populations (1). During the COVID-19 pandemic there was a further increase in youth-onset T2D, likely due to more sedentary behaviors (2). Family history of diabetes, quality of and access to health care, differences by race/ethnicity in insulin sensitivity, diet quality, and minimal physical activity are some of the risk factors for T2D (3). Furthermore, T2D in youth has been found to be more aggressive in nature with rapid deterioration in β -cell functioning and high rates of diabetes complications, compared to T2D in adults (4, 5). Given the clinical course of youth-onset T2D and limited treatment options (metformin, insulin or subcutaneous GLP-1 agonist), there is an increasing need for interventions that not only improve glycemic control and reduce insulin resistance, but also promote behavioral modifications such as exercise and healthy eating.

In youth with type 1 diabetes (T1D), the use of continuous glucose monitoring (CGM) has been associated with decreased hemoglobin A1c (HbA1c) levels and increased time in range (TIR), while also improving quality of life (6, 7). Similar trends have been noted in adults with T2D, in which investigators concluded that the real-time glycemic feedback provided by CGM gave way to lifestyle modifications, including more physical activity, that led to improvements in glycemic control (8, 9). The SEARCH study found that most youth with T2D test their glucose levels fewer than 3 times per day (8) and 27% of youth with T2D had poor glycemic control (HbA1c > 9.5%) (10–12).

Given the improvements observed when implementing CGM in youth with T1D and adults with T2D, there is reason to believe that CGM could play a pivotal role in helping youth with T2D check their blood sugars more frequently, while also promoting behavioral modifications that improve glycemic control. There is limited information on the use of CGM in youth with T2D. We conducted a pilot clinical trial to determine if a complementary 10-day trial of CGM use in youth with T2D impacts glycemic control and behavior.

## 2 Methods

This was a prospective, interventional, single-group assignment trial that was conducted at the Johns Hopkins Pediatric Diabetes Center at two sites (Johns Hopkins Hospital and Mount Washington Pediatric Hospital) in Baltimore, Maryland. The Johns Hopkins Medicine Institutional Review Boards reviewed and approved the study protocol. Assent from participants and consent from the parent or legal guardians was obtained prior to enrollment. The enrollment period was January 20, 2021 to February 15, 2022, with the final study follow-up visit on July 28, 2022. This study was preregistered at ClinicalTrials.gov (NCT04721158).

### 2.1 Study participants

Participants ages 8 to 21 diagnosed with T2D for >3 months and no CGM use in the past 12 months were eligible to participate in the study. Participants with T2D not on insulin were excluded.

### 2.2 Study design

Eligible participants were approached during in-person, routine diabetes care visits, and offered the opportunity to enroll in the study. Following patient consent, a diabetes provider placed the CGM. A GrifGrip adhesive was applied over the CGM to secure the device on the skin.

The diabetes educator provided CGM education which took approximately 5 minutes. Education included target blood sugar range of 70-180 mg/dL, use of trend arrows, intervention for alerts (high alert set at 300 mg/dL, low alert set at 70 mg/dL) and education on Dexcom app. The Dexcom G6 and Dexcom Clarity apps were set up on the participant’s or caregiver’s compatible smartphone. If the patient did not have a compatible smartphone, a loaner phone was offered for use during the 10-day trial period. Clinic sharing was set up during enrollment.

At the time of enrollment, demographic information (data of birth; gender/sex; race; insurance information; highest parental education level; and household income) and information on diabetes care (date of diabetes diagnosis; age of diagnosis; medications including insulin, metformin or liraglutide) was recorded. HbA1c was recorded as part of routine procedures. During this visit, a research coordinator assessed current blood sugar (BG) monitoring and diabetes distress through behavioral questionnaires administered via an iPad that was linked to RedCap (13, 14): Perceived Benefits of CGM Scale (BenCGM) (15), Perceived Barriers of CGM Scale (BurCGM) (15), The Glucose Monitoring Satisfaction Survey Type 2 Diabetes (GMSS-T2D) (16), Diabetes Distress Scale (DDS) (17), and Pediatric Quality of Life 3.2 Diabetes Module (PedsQL) (18). Of note, one of the questions from the BenCGM was inadvertently excluded from the RedCap surveys (19). For participation in the trial, participants received a parking voucher and $10 gift card. After the initial visit, all participants had follow-up visits via phone or video call (based on participant preference) after 5 and 10 days, and in-person at 3-6 months, coinciding with their regularly scheduled diabetes care visit. The 5- and 10-day follow-up call timing and phone numbers were confirmed with the participant during the enrollment visit, and 3-month follow up visits were scheduled at the time of enrollment.

At the 5-day (4-6 days if 5^th^ day fell over a weekend) and 10-day follow-up visit, the diabetes educator called the patient and recorded 5-day average glucose reading and time in range (TIR) data. The diabetes educator asked questions regarding CGM use and behaviors. Dose adjustments and education on obtaining a personal CGM were provided. After the 10-day follow-up call, the same surveys at enrollment were emailed to participants to complete.

The 3-month follow-up visit was conducted in person as part of routine diabetes care. HbA1c was recorded as part of routine standard of care using the Afinion AS100 analyzer. Furthermore, use of CGM was noted and where possible, CGM data, including average glucose (mg/dl), time in range (scale: very high, high, in-range, low, very low) (%), and days with CGM data (% days) were collected. Participants were again administered surveys through RedCap via an iPad. Participants received a second parking voucher and $10 gift card. If the participant did not attend the 3-month visit, attempts were made to reschedule the visit. If the participant did not show for the scheduled visits, research staff attempted to contact the participant via telephone to ask follow-up questions, and surveys were emailed to participants. Another attempt was made to collect follow-up data at the 6-month visit. If surveys were not completed by the next in-person 6-month follow-up visit, they were recorded as incomplete.

### 2.3 Outcomes

The primary outcomes of the study were change in TIR from the first 5 days compared to the second 5 days of CGM wear, and change in HbA1c level from baseline to follow up at 3-6 months. Secondary endpoints included behavioral modification assessed via questions during 5-day and 10-day follow up calls and responses to behavioral surveys.

### 2.4 Sample size calculation

We calculated the sample size to detect a 10% difference in TIR from the first 5-days compared to the second 5 days of CGM wear. Assuming a TIR SD of 18% (20), a 2-sided alpha level of 0.05 and 80% power, a sample size of 28 participants was required. Allowing for 30% attrition, we planned to recruit 40 participants.

### 2.5 Statistical methods

Categorical variables are described using frequencies and percentages. The Shapiro-Wilks test was used to assess the normality distribution of continuous variables. Normally distributed variables were described by mean and standard deviation. Non-normally distributed variables were described by median and interquartile range. Paired t-tests were used to assess most differences between baseline and follow up. Wilcoxon signed rank tests were used for the Diabetes Distress Scale scores and sub-scores. Time in range data from the first 5 ( ± 1) days of CGM wear was compared to the second 5 ( ± 1) days for any patients with at least 8+ days of CGM wear. Additional comparisons were made with the follow-up CGM data recorded at the 3 or 6-month follow-up. Three month follow up data was used when available. For the n=13 patients missing 3-month visits, but with later follow-up, the 6-month visit was used. Two sample t-tests, and Wilcoxon rank sum tests (for the Diabetes Distress Scale), were used to assess differences between groups in survey responses. The values p< 0.05 were considered statistically significant. Statistical analysis was generated using SAS version 9.4 Copyright ^©^ 2020 SAS Institute Inc., Cary, NC.

## 3 Results

### 3.1 Participant demographics and clinical characteristics

There were 41 youth ages 11-18 years enrolled with median age of 16.2 years (IQR 15.0-17.5), 61% female, 81% Non-Hispanic (NH) black, median diabetes duration of 0.8 years (IQR 0.4-2.6), baseline median HbA1c of 10.3% (7.3-12.5) and median BMI 37.3 kg/m^2^ (30.8-46.1). A majority had public insurance (76%), household income<$50K (81%) and parental education level of HS degree or less (73%). The study required that participants were treated with insulin and 40/41 participants were on insulin prior to study start, and one patient was started on insulin at the same visit as enrollment. A majority of participants (85%) were on basal/bolus insulin and (80.5%) metformin with a smaller percentage (4.9%) on liraglutide. Of note, 11 (26.8%) participants needed a loaner compatible smart phone in order to use the CGM device (see **Table 1**).

**Table 1:** Participant demographic information.

Characteristic	N (%)
Age (years), median (IQR)	16.2 (15.0-17.5)
Sex, male	16 (39%)
Race/ethnicity	
NH White	4 (9.8%)
NH Black	33 (80.5%)
Hispanic	4 (9.8%)
Public Insurance	31 (75.6%)
Parent Education (n=40)	
<HS	4 (10%)
HS	25 (62.5%)
>HS	11 (27.5%)
Parental Income<$50,000 (n=32)	26 (81.3%)
Duration of DM (years), median (IQR)	0.8 (0.4-2.6)
Diagnosis Age (years), mean (SD)	14.2 (2)
HbA1c percentage, median (IQR)	10.3 (7.3-12.5)
Body Mass Index (BMI – kg/m^2^), median (IQR)	37.3 (30.8-46.1)
On insulin	40 (97.6%)
Basal/bolus regimen	34 (85%)
Insulin dose (units/kg/day), median (IQR)	0.48 (0.33-0.8)
On Metformin	33 (80.5%)
Metformin dose (mg/day), median (IQR)	2000 (1925-2000)
On Liraglutide	2 (4.9%)
Needed compatible smart phone to use CGM	11 (26.8%)

**Figure 1:**
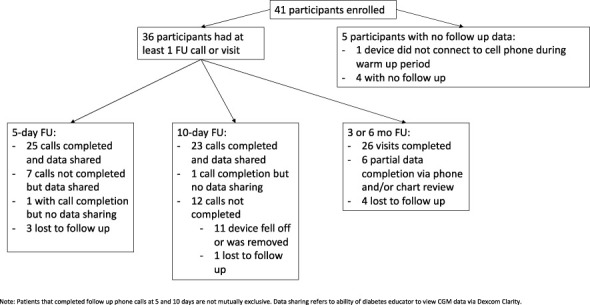
Flowchart of participant enrollment and participation.

As seen in **Figure 1**, 5 patients enrolled but did not complete any part of the study past enrollment either due to device failed to connect to smart phone during 2-hour warm-up period (1) or lost to follow-up (4). There were 25 participants who completed the 5-day follow-up call and CGM data was viewed by the clinic. There were 23 participants who completed the 10-day follow-up call and data was viewed by the clinic, and 26 participants who completed either the 3 and/or 6 month follow-up visits (see **Figure 1**). Of the participants who completed 3 and/or 6 month follow-up, the median BMI was 35.8 kg/m^2^ (IQR 30.1-39.3).

### 3.2 CGM use and relationship to glycemic outcomes

Among the 30 participants that completed 3 or 6-month follow up, mean baseline HbA1c was 10.2% and at the 3-month follow up the mean HbA1c was 10.3% (*p*=0.89). Of the 19 participants who completed at least 8 days of the 10-day trial CGM, average 5-day TIR was 49.2% and the average10-day TIR was 50.7% (*p*= 0.62); average 5-day glucose was 213 mg/dL vs. 212 mg/dL at 10 days *(p*=0.86*).* Only 2 participants were using a Dexcom CGM with remote access by providers to their data at 3-6 month follow up. There was insufficient Dexcom uptake at 3- and 6- month follow-up visits to compare trial-CGM TIR or glucose data to 3-6 month follow-up data. Three additional patients reported that they were using Freestyle Libre CGM at follow-up, and one additional patient reported Dexcom CGM use but remote access to their data was unavailable.

Of note, two of the enrolled participants presented in diabetic ketoacidosis with HbA1c>14% within one month after the 10-day CGM trial. One participant developed a skin infection at CGM insertion site, despite cleaning skin with alcohol pad prior to insertion. The infection resolved after device removal.

### 3.3 Qualitative results

At the 5-day and 10-day follow up calls, the diabetes educator asked participants questions regarding CGM use. **Table 2** lists the questions asked of participants as well as participant responses. A majority of participants reported positive behavioral changes including giving insulin more often, checking BG more often, avoiding high sugar foods, and exercising more. The diabetes provider made insulin dose changes based on the data provided by the participant during follow-up calls.

**Table 2:** Participant responses to 10-day follow up call questions.

Question	Participant Responses (n)
What is going well? (Patient could provide multiple responses)	More insulin (4) More BG checks (19) Avoid high sugar foods (5) Change other habits (2) See the effect of eating on BG (3) Avoiding fingersticks/pricking (3) Likes alerts (2)
Do you use arrow to make treatment decisions?	Yes (16) No (6) Sometimes (2)
Do you look at or check your blood sugar more often than before?	Yes (21) No (0) Sometimes (1)
Did you take your insulin more often than before?	Yes (14) No (8) Sometimes (2)
Did you have any severe highs?	Yes (14) No (10)
Did you have any severe lows?	Yes (1) No (21)
Based on time in range data, asked “What days went well?” “How can that be replicated?”	Taking insulin more often (3) Checking BGs more often (6) Avoiding foods that cause higher blood sugars (8) Unable to identify (9)
Areas to improve: Any patterns identified related to foods, dosing and behaviors?	More BG checks (6) Insulin before meals (7) Avoid high sugar foods (4) Exercise (6) Reduce snacking (1) Limit portion sizes (2) Take insulin as prescribed (2)
Insulin dose change?	Yes (13) No (10)
Would you like to get a personal CGM?	Yes (19) No (4) Maybe (1)

Based on participant report, 95.8% reported increased BG checks, 58.3% reported more insulin usage and 54.2% had insulin doses adjusted after 10 days of CGM use. 96% of participants felt that the CGM improved their diabetes management at 3-6 month follow-up visits.

### 3.4 Survey results

Participants were asked to complete the following surveys at the initial visit, 10-day follow up call and 3-6 month follow up visit: BenCGM (15), BurCGM (15), GMSS-T2D (16), DDS (17), and PedsQL (18).

Other than a slight increase in the diabetes interpersonal distress scale among the 18 participants completing both baseline and 3-6 month DDS surveys (*p*=0.03), there was no statistically significant difference in any of the survey scores obtained at baseline when compared to 10-day or at 3-6 month follow up (See **Table 3**).

**Table 3:** Comparison of behavioral questionnaires at baseline visit to 10 day and 3 month follow up.

Survey	Baseline		10 Days			3 months		
	N	Mean (SD)	N	Mean (SD)	p-value^b^	N	Mean (SD)	p-value^b^
Perceived Benefits of CGM Scale (BenCGM)	41	4.1 (0.7)	23	4.1 (1)	0.67	20	3.8 (1)	0.25
Perceived Barriers of CGM Scale (BurCGM)	41	1.95 (0.7)	23	1.8 (0.7)	0.40	20	1.97 (0.8)	0.76
PedsQL 3.2 Diabetes Module	40	71.1 (15.9)	22	70.3 (17.3)	0.61	20	70.5 (15.9)	0.51
Diabetes Distress Scale (DDS)a	37	1.9 (1.2-2.5)	23	1.4 (1-2.6)	0.68	20	1.8 (1.2-2.6)	0.64
DDS - Emotional burden subscore	37	2 (1.2-3.6)	23	1.6 (1-2.6)	0.89	20	1.7 (1.1-3.5)	0.79
DDS - Physician related distress	37	1 (1-2.3)	23	1 (1-1)	0.25	20	1 (1-2)	0.37
DDS - Regiment related distress	37	1.6 (1.2-2.8)	23	1.4 (1-3.6)	0.19	20	2.3 (1.3-3.5)	0.21
DDS Interpersonal Distress	37	1 (1-1.7)	23	1 (1-1.7)	0.18	20	1 (1-1.5)	**0.031**
The Glucose Monitoring Satisfaction Survey Type 2 Diabetes (GMSS-T2D)	39	3.8 (0.6)	22	3.8 (0.6)	0.69	19	3.4 (0.6)	0.24
GMSS - Openness	41	3.4 (0.7)	23	3.1 (0.9)	0.12	20	3 (0.8)	0.43
GMSS - Emotional burden	41	2.2 (0.8)	23	2.2 (0.8)	0.38	20	2.6 (0.8)	0.051
GMSS - Behavioral burden	41	2 (0.8)	23	1.8 (0.8)	0.49	20	2.1 (0.7)	0.87
GMSS - Worth	39	3.9 (0.7)	22	4.1 (0.8)	0.34	19	3.5 (0.8)	0.35

a DDS scores are not normally distributed. Median and IQR are reported.

b Paired T-tests are used to calculate p values for all surveys except DDS and DDS sub-scales which use the Wilcoxon signed-rank test. Tests are conducted on subjects with both baseline and follow completed. The bolded value is statistically significant p-value with p-value < 0.05.

## 4 Discussion

In this pilot clinical trial providing complementary 10-day CGM use to youth with T2D, we demonstrated that CGM use is associated with behavioral modifications, where adolescents checked BG more frequently, gave insulin more frequently and endorsed a general sense that CGM improved diabetes management. Additionally, a majority of participants wanted to continue using a personal CGM following the trial. However, there was no improvement in CGM TIR or change in HbA1c level from baseline to follow up at 3-6 months and survey results showed that CGM use did not change quality of life or overall diabetes distress.

There is limited data available on the use of CGM in youth with T2D. The SWITCH and CITY studies in children with T1D showed a mean reduction in HbA1c by 0.4% (22, 23) with long-term CGM use. In studies of adults with T2D, CGM use was associated with mean reduction in HbA1c of 0.3-0.5% (21, 24, 25). Additionally, a recent study of young adults with T2D suggested that glucose variability may portend worse outcomes (26), such that CGM use could be helpful by enabling providers to detect glucose variability before noticeable changes in HbA1c, allowing more time for clinical intervention (26). While we did not see increased time in range during the 10-day CGM study period, it is possible there was a decrease in glycemic variability without a change in TIR. Additionally, the 10-day CGM trial may have been too brief, and future studies should consider longer CGM trial periods.

Studies in adults with T2D have shown increased physical activity and improved body composition with CGM use (9, 27, 28). Participants in our short-term trial endorsed similar behavioral improvements including checking BG more often and administering insulin more frequently. If these positive changes were to continue with long-term use of CGM, then participants may see long-term improvement in diabetes management that could potentially mitigate the high risk for long-term complications in youth-onset T2D (4). Future studies should examine the potential for long-term CGM use to increase lifestyle modifications with more physical activity and optimal dietary choices.

Our study population was mostly composed of minority youth, similar to the TODAY study of youth onset T2D, but unlike the aforementioned studies of CGM use in youth with T1D and adults with T2D (9, 22, 23, 27–29). Our baseline HbA1c 10.3% is higher than those seen in any of the adult referenced studies where baseline HbA1c levels were approximately 8.5% (9, 27, 28), and higher than patients enrolled in the TODAY study where baseline HbA1c was 5.9%, and this is likely attributed to our study inclusion criteria in which participants had to be on insulin therapy. In addition, our study was intended to target youth with poor glycemic control.

Our study population was also representative of the low socioeconomic status seen in youth with T2D. Data from the TODAY trial demonstrated that 25% of parents’ highest level of education was less than HS, and 40% of families had a yearly family income of ≤ 5K (29). In the Pediatric Diabetes Consortium clinic registry, 70% of parents of children with T2D had obtained a HS education or less and 43% had a yearly family income of ≤ . $K (30). Similarly, in our study population, 73% of parents’ education was high school degree or less and 80% had a yearly family income of ≤ 0K. Studies have shown that low income leads to reduced access to diabetes care due to issues with transportation or taking time off from work, and as a result suboptimal glycemic control (31).

In this trial we encountered several obstacles in establishing follow-up with participants. Although participants were given 3-month follow-up appointments at enrollment, many did not attend or rescheduled their follow-up visits. Additionally, it was difficult to reach participants via telephone, despite confirming a date/time that worked best for parent/participant during the initial study visit. Multiple phone numbers were obtained in order to improve communication with the study team. Unfortunately, the obstacles that we encountered in following up with participants are common in adolescents with T2D. A study by the Pediatric Diabetes Consortium found that 55% of patients were lost to follow-up after a median of 1.3 years (from enrollment to the last visit date), which was similar to our in-person clinic follow up rate of 59% (32).

Interestingly, a majority of participants wanted a CGM at the conclusion of the study. Of the 14 patients who did not complete the 10-day trial wear, 8 were due to sensor or adhesive issues, with the device falling off early despite application of GrifGrips. This is a known barrier to CGM usage in children with diabetes (33). While none of the participants described alarm fatigue as a barrier to CGM use, many of the participants had HgbA1c > 10% and thus had average blood sugars of at least 240 mg/dL. Given that the CGM high alert was set at 300 mg/dL, it is possible that the lack of improvement in glycemic control was partly attributed to the constant high BG reminders which possibly resulted in ignoring alarms instead of prompting insulin administration or lifestyle changes.

Study limitations include the small study size with a limited number of participants completing both 10-day trial period and 3/6-month follow up visits. There were a large number of patients who had device connectivity and adhesive issues. Additionally, as discussed above many participants were lost to follow-up. The primary strength of this study is that, to our knowledge, it is the first study to evaluate CGM use and relationship to behavioral/lifestyle choices and glycemic control in adolescents with T2D.

## 5 Conclusions

Although 10-day trial CGM use did not impact short-term or long-term glycemic control in this sample of youth with T2D, most participants reported positive behavioral changes. Of those who completed the full 10 days of CGM use, most wanted to continue using CGM. Issues with device connectivity and adhesion were barriers to consistent device use. Future larger studies with longer use of CGM may help clarify the potential impact of CGM in youth with T2D.

## Data availability statement

The raw data supporting the conclusions of this article will be made available by authors to bona fide researchers in deidentified/anonymized format, without undue reservation.

## Ethics statement

The studies involving human participants were reviewed and approved by Johns Hopkins IRB. Written informed consent to participate in this study was provided by the participant (if 18 years or older) or by the participant's legal guardian/next of kin (if under 18 years old).

## Author contributions

RMW and SNM conceptualized the study. TL and JM recruited the participants, RG, KA, and TL completed the data collection, EAB performed the analysis, JM, RG, KA, EAB, and RMW wrote the manuscript, and all authors critically reviewed the final draft. RMW is the guarantor of this work and, as such, had full access to all the data in the study and takes responsibility for the integrity of the data and the accuracy of the data analysis. All authors contributed to the article and approved the submitted version.
