# Supplementary material for: Short-term use of CGM in youth onset type 2 diabetes is associated with behavioral modifications
Source: Front Endocrinol (Lausanne). 2023 May 29;14:1182260. doi: 10.3389/fendo.2023.1182260 (PMC10258317; doi:10.3389/fendo.2023.1182260)
Supplement: Supplementary file 1 [file Table_1.pdf]

Supplemental Table 1: Comparison of Glycemic Data

	**Baseline**	**5 Days**	**10 Days**	**3-6 months**
Variable	Mean (SD)	Mean (SD)	Mean (SD)	Mean (SD)
HbA1c (%), n=30	10.2 (2.8)	-	-	10.3 (3.2)
Glucose (mg/dL), n=18	-	213 (79)	212 (92)	-
Time in Range 70-180 mg/dL (%), n=18	-	49.2% (38)	50.7% (39)	-
Time Below Range < 70 mg/dL (%), n=18	-	0.1% (0.2)	0.1% (0.5)	-
Time Above Range > 180 mg/dL (%), n=18	-	50.6% (37.8)	49.1% (39.6)	-

Note: Only includes participants with baseline and follow up values who wore CGM for at least 8 days
